# Engineered Resistant-Starch (ERS) Diet Shapes Colon Microbiota Profile in Parallel with the Retardation of Tumor Growth in In Vitro and In Vivo Pancreatic Cancer Models

**DOI:** 10.3390/nu9040331

**Published:** 2017-03-27

**Authors:** Concetta Panebianco, Kaarel Adamberg, Signe Adamberg, Chiara Saracino, Madis Jaagura, Kaia Kolk, Anna Grazia Di Chio, Paolo Graziano, Raivo Vilu, Valerio Pazienza

**Affiliations:** 1 Gastroenterology Unit, I.R.C.C.S. “Casa Sollievo della Sofferenza”, Hospital, San Giovanni Rotondo (FG) 71013, Italy; panebianco.c@gmail.com (C.P.); chiarasaracino88@gmail.com (C.S.); 2 Department of Food Processing, Tallinn University of Technology, Tallinn 12616, Estonia; kaarel.adamberg@ttu.ee (K.A.); signe.adamberg@gmail.com (S.A.); 3 Competence Center of Food and Fermentation Technologies, Tallinn 12616, Estonia; madis.jaagura@ttu.ee (M.J.); raivo@kbfi.ee (R.V.); 4 Department of Chemistry, Tallinn University of Technology, Tallinn 12616, Estonia; kaia.kolk@ttu.ee; 5 Tamma Industrie Alimentari di Capitanata (FG), Foggia 71100, Italy; adichio@tamma.it; 6 Pathology Unit, I.R.C.C.S. “Casa Sollievo della Sofferenza”, Hospital, San Giovanni Rotondo (FG) 71013, Italy; p.graziano@operapadrepio.it

**Keywords:** pancreatic cancer, microbiota, functional food

## Abstract

Background/aims: Pancreatic cancer (PC) is ranked as the fourth leading cause of cancer-related deaths worldwide. Despite recent advances in treatment options, a modest impact on the outcome of the disease is observed so far. We have previously demonstrated that short-term fasting cycles have the potential to improve the efficacy of chemotherapy against PC. The aim of this study was to assess the effect of an engineered resistant-starch (ERS) mimicking diet on the growth of cancer cell lines in vitro, on the composition of fecal microbiota, and on tumor growth in an in vivo pancreatic cancer mouse xenograft model. Materials and Methods: BxPC-3, MIA PaCa-2 and PANC-1 cells were cultured in the control, and in the ERS-mimicking diet culturing condition, to evaluate tumor growth and proliferation pathways. Pancreatic cancer xenograft mice were subjected to an ERS diet to assess tumor volume and weight as compared to mice fed with a control diet. The composition and activity of fecal microbiota were further analyzed in growth experiments by isothermal microcalorimetry. Results: Pancreatic cancer cells cultured in an ERS diet-mimicking medium showed decreased levels of phospho-ERK1/2 (extracellular signal-regulated kinase proteins) and phospho-mTOR (mammalian target of rapamycin) levels, as compared to those cultured in standard medium. Consistently, xenograft pancreatic cancer mice subjected to an ERS diet displayed significant retardation in tumor growth. In in vitro growth experiments, the fecal microbial cultures from mice fed with an ERS diet showed enhanced growth on residual substrates, higher production of formate and lactate, and decreased amounts of propionate, compared to fecal microbiota from mice fed with the control diet. Conclusion: A positive effect of the ERS diet on composition and metabolism of mouse fecal microbiota shown in vitro is associated with the decrease of tumor progression in the in vivo PC xenograft mouse model. These results suggest that engineered dietary interventions could be supportive as a synergistic approach to enhance the efficacy of existing cancer treatments in pancreatic cancer patients.

## 1. Introduction

As the fourth leading cause of death for cancer worldwide, adenocarcinoma of the pancreas is a highly lethal tumor [1]. Surgical resection is the only curative treatment option, but unfortunately only a small percentage of patients are eligible because diagnosis often occurs late, when the disease is at an advanced stage [2]. Poor survival rates are also due to cancer aggressiveness and chemo-resistance, which make existing systemic therapies ineffective. Despite both intrinsic and acquired resistance mechanisms that decrease drug efficacy [3], gemcitabine, alone or in combination with other drugs, has long been considered the first-line option in the therapy of pancreatic cancer (PC) [4]. Our recent breakthrough studies uncovered a potential link between short cycles of fasting and improved effectiveness of chemotherapy [5]. Specifically, short-term (24 h) starvation achieved with fasting-mimicking medium increases the uptake of gemcitabine by tumor cells, rendering them more susceptible to drug-induced cell death. Consistently, in an in vivo model of xenograft pancreatic cancer, gemcitabine administered to 24 h-fasted mice significantly decreased tumor volume, as compared to control mice [5]. In addition, fasting-mimicking medium was shown to shift cells to the G0/G1 phase of the cell cycle. Consistently, fasting cycles decrease the levels of the proliferation marker Ki67 in vivo [5], in agreement with the finding that caloric restriction decreases murine and human pancreatic cell growth [6]. In light of these observations, fasting could also reduce cancer growth and increase the effectiveness of chemotherapy in patients with PC. However, shifting this therapeutic approach from animals to humans has to overcome some objective difficulties: diseased people may refuse to follow the fasting regimen [7], and fasting may worsen the weight loss that often occurs in cancer patients. Therefore, alternative approaches are needed, to take advantage of the benefits of caloric restriction without requiring special waivers from patients. In this regard, we focused on dietary carbohydrates, recognized as pivotal elements in the metabolism of cancer cells, and as promoters of cancer growth [8]. Reports on the role of a low-carbohydrate diet in reducing tumor growth already exist, but in these diets, a higher content of protein or fat was supplied as an alternative energy source [9,10]. Conversely, we formulated an engineered resistant-starch (ERS) diet in which corn starch was replaced by resistant starch. While common starch is metabolized by the enzymes of the small intestine to release glucose, resistant starch is not digestible, so it reaches the large intestine where it is fermented by resident bacteria, to produce bioactive metabolites such as short chain fatty acids (acetate, propionate, butyrate, valerate), other organic acids (lactate, succinate and formate), gases, and alcohols [11]. It is known that diet can shape the composition of the gut microbiota [12,13,14], whose alterations are increasingly emerging as a key factor in the development of gastrointestinal diseases and metabolic disorders [15,16], as well as of either intestinal and extra-intestinal cancers [17,18]. Several studies have revealed a link between alterations in oral and gut microbiota composition, and the development of pancreatic cancer, which is likely due to the ability of certain bacterial populations to sustain inflammation, which in turn promotes cancer [19,20,21].

In the current study, we sought to assess whether dietary interventions replacing corn starch with resistant starch may be beneficial in reducing tumor growth, in an animal model of xenografted PC, and to determine the molecular and microbial profile during tumor development.

## 2. Materials and Methods

### 2.1. Cell Culture and ERS-Mimicking Condition (EMM)

MIA PaCa-2 cells were cultured either in control DMEM medium (CM): 2 g/L glucose supplemented with 10% fetal bovine serum (FBS), 100 U/mL penicillin and 100 μg/mL streptomycin (Invitrogen Life Technologies, Milan, Italy) in 5% CO_2_ atmosphere at 37 °C, or in ERS-mimicking medium (EMM): DMEM (0.5 g/L glucose and 1% FBS). BxPC-3, and PANC-1 were maintained in control Roswell Park Memorial Institute medium (RPMI, Invitrogen Life Technologies, Milan, Italy), or in ERS-mimicking condition RPMI medium as described elsewhere [22].

### 2.2. Animal Study

The in vivo study was performed in an Association for Assessment and Accreditation of Laboratory Animal Care International (AAALAC)-accredited experimental facility. Animal protocols were approved by the Institutional Animal Care and Use Committee with the approval number *ANM14-002*. 5 × 10^6^ BxPC-3-luc cancer cells per mouse were suspended in 0.1 mL of PBS/matrigel mixture (1:1) and then subcutaneous (s.c.) injected (right flank) into 5–6 weeks old female Nu/Nu nude mice. When tumor size reached an average volume of 100 mm^3^, BxPC-3-luc tumor-bearing nude mice were randomly assigned into two groups (six mice/group): Group 1 (under standard diet) and Group 2 (under ERS diet). Animals had free access to water. The ERS diet pellets had corn starch replaced entirely with resistant starch (Hi Maize 260).

Fresh fecal samples were collected before and after cancer induction from both feeding groups. The fecal samples were collected into a regular sterile 1.5 mL Eppendorf and kept frozen at −80 °C until use in cultivation experiments.

### 2.3. Immunoblotting

Total protein extractions from pancreatic cancer adherent cells and from snap-frozen pancreatic cancer xenograft specimens were obtained using homemade Sample Buffer Laemmli 2× (50 mM Tris–HCl, pH 6.8, 100 mM dithiothreitol, 2% sodium dodecyl sulfate, 0.1% bromophenol blue, 10% glycerol) supplemented with 2× protease inhibitor cocktail (COMPLETE; Roche Diagnostics, Mannheim, Germany), 1 mM phenylmethylsulphonyl fluoride, and 1 mM sodium orthovanadate as previously described [23]. Equal amounts of protein extract for each sample was loaded onto 10% SDS-polyacrylamide gel and transferred on PVDF membrane (Whatman, Dassel, Germany) for 60 min at 60 V. Membranes were incubated overnight at 4 °C with primary antibody diluted 1:1000 into Blocking Buffer as reported in [23]. Primary mouse monoclonal antibodies against β-Actin (C4) (sc-47778) was sourced from Santa Cruz Biotechnology (D.B.A., Milan, Italy); antibodies against ERK1/2 (#4370), phospho ERK1/2 (#4695), mTOR (#2972), phospho-mTOR (#2974), p70S6K (#9202), phospho-p70S6K (#9205) were obtained from Cell Signaling. The membranes were washed three times with washing solution (1× Tris-Buffered Saline, 0.1% Tween 20 Sigma) and then incubated for one hour at room temperature with appropriate secondary antibodies (Bio-Rad, Hercules, CA, USA, goat anti-mouse and goat-anti-rabbit diluted 1:3000). Detection of the antigen-antibody complexes on X-ray film (Amersham Biosciences) was performed using enhanced chemiluminescence (ECL; Amersham Biosciences) according to the manufacturer’s instructions.

### 2.4. Proliferation Assay

The proliferation of cells cultured in either control medium or EMM for 0 h, 24 h or 48 h was assessed as follows: cells were trypsinized, resuspended in complete medium, and incubated for five minutes with the Muse Count & Viability Reagent (Merck Millipore, Milan, Italy), according to the manufacturer’s instructions and then ran on Muse Cell Analyzer (Merck Millipore, Milan, Italy).

### 2.5. Immunohistochemistry

Paraffin-embedded pancreatic mice cancer sections allocated into the two different groups were immunostained by using a commercially available detection kit (EnVision™ FLEX+, Dako, Glostrup, Denmark), following the manufacturer’s protocol previously described [24]. Primary antibodies for Ki67 (cat. No. M7240) were from Dako. The primary antibody was replaced with normal serum alone to check the specificity of all reactions. Positive and negative controls were run concomitantly. Ki67 immunoreactivity was evaluated blindly by an expert pathologist assessing a semi-quantitative scoring system in ten high power fields (10 HPF, X 400) according to a semi-quantitative scale from negative to 3+ (−: 0%; +: 1%–33%; ++: 34%–66%; +++: 67%–100%).

### 2.6. Quantitative Real-Time Polymerase Chain Reaction

Total RNA was extracted from snap-frozen pancreatic cancer xenograft biopsies using the Qiazol Lysis Reagent (Qiagen, Milan, Italy), according to the manufacturer’s instructions. RNA concentration and quality were assessed using NanoDrop spectrophotometer. Quantitative real-time PCR (qRT-PCR) for measuring Ki67 expression levels was performed on 50 ng of purified RNA using the one-step Quantifast SYBR Green RT-PCR KIT (Qiagen) and the primers 5′-CAGCTTAAGGGAGGCTTCTT-3′ (forward primer) and 5′-GTAACCAGGAAATGCAGTCG-3′ (reverse primer). Reactions were set up in 384-well plates using a 7900HT Real-Time PCR System (Applied Biosystems, Foster City, CA, USA), and all samples were assayed in triplicate. Optical data obtained were analyzed using the default and variable parameters available in the SDS software package (version 2.4; Applied Biosystems, Foster City, CA, USA). Expression levels of Ki67 were normalized to the housekeeping gene TATA binding protein (TBP, Qiagen, QT00000721). mRNA amount of Ki67 relative to TBP was calculated through the 2^(−ΔΔCt)^ method. Data are presented as mean ± standard deviation (SD).

### 2.7. Isothermal Microcalorimetry

For inocula preparation, 0.02–0.4 g fecal samples were thawed and mixed with five sterile deaerated phosphate-buffered saline (PBS)- containing (final concentration, mM): NaCl-160, KCl-3, Na_2_HPO_4_-8, NaH_2_PO_4_-1, pH 7.2, supplemented with freshly made and filter-sterilized Cys-HCl (0.5 g/L in final medium), a solution of autoclaved sodium thioglycolate (0.5 g/L in final medium) as a reducing agent, and 4 mL substrate solution or water (as a control without additional carbohydrates). Substrate solution contained 5 g/L of levan prepared as shown in [25], or contained resistant starch (Tapioca maltodextrin, C1, Cargill Germany Gmbh, Krefeld, Germany). All growth media were pre-reduced in an anaerobic jar (Anaero-Gen™, GasPack System, Oxoid, Inc., Basingstoke, United Kingdom) before inoculation of the fecal cultures.

The 3.3 mL calorimeter ampoules were filled with 2 mL of the inoculated medium, closed hermetically, and incubated at 37 °C in a 24-channel isothermal microcalorimeter TAM III (TA Instruments, New Castle, DE, USA) as described in Kabanova et al. [26]. The heat flow (P, μW) was recorded and total heat accumulated (Q, J) proportional to biomass amount was calculated by integration of heat flow. All fecal samples were tested at least in duplicate.

### 2.8. Determination of Metabolites

Samples from the beginning and end of the growth experiments were analyzed for microbial 16S rDNA sequences and metabolites. The samples were centrifuged (21,000× *g*, 10 min), a solution of 10% sulfosalicylic acid was added to the supernatant (1:0.25 vol/vol), and both pellet and supernatant stored at −20 °C until the analysis. Before chromatographic analyses, the supernatant samples were centrifuged (21,000× *g*, 15 min, 4 °C) and filtered through 0.20 μm Polytetrafluoroethylene PTFE syringe filters (Millex filters SLLGH13NK, Millipore, Tallinn Estonia). The initial (0 h) samples were additionally ultra-filtered using AmiconR Ultra-10K Centrifugal Filter Devices, 10 kDa cut-off (Millipore).

The concentrations of organic acids (succinate, lactate, formate, acetate, propionate, isobutyrate, butyrate, isovalerate, valerate), glycerol and ethanol were determined by high-performance liquid chromatography (HPLC, Alliance 2795 system, Waters, Milford, MA, USA), using a Bio-Rad HPX-87H column (Bio-Rad Laboratories, Hercules, CA, USA) with isocratic elution of 0.005 M H_2_SO_4_ at a flow rate of 0.5–0.6 mL/min at 35 °C. Refractive index (RI) (model 2414; Waters) and UV (210 nm; model 2487; Waters) detectors were used for quantification of the substances. Detection limit for the HPLC was 0.1 mM.

### 2.9. Microbiome Analyses

DNA was extracted from the cell pellet using MoBioPowerFecal DNA extraction kits (MoBio, Carlsbad, ON, Canada) according to the manufacturer’s instructions. Universal primers S-D-Bact-0341-b-S-17 Forward 5′ TCGTCGGCAGCGTCAGATGTGTATAAGAGACAGCCTACGGGNGGCWGCAG

F and S-D-Bact-0785-a-A-21 Reverse 5′ GTCTCGTGGGCTCGGAGATGTGTATAAGAGACAGGACTACHVGGGTATCTAATCC were used for PCR amplification of the V3-V4 hypervariable regions of 16S rRNA genes [27]. The amplified region was about 450 bp and on average, 12,000 reads per sample were obtained. The mixture of amplicons was pyrosequenced using Illumina MiSeq 2 × 250 v2 platform.

Sequence data were analyzed using BION-meta, an open source program, according to author’s instructions. First, sequences were trimmed at both ends using a cut-off for minimum quality of 95%, followed by removal of reads shorter than 350 bp. Secondly, sequences were clustered based on a minimum seed similarity of 99.5% (consensus reads). Lastly, consensus reads were taxonomically aligned to the SILVA reference 16S rDNA database (v123) using a match minimum of 90%.

### 2.10. Statistical Analysis

Shannon diversity index (*H’*) was calculated according to formula: $H\prime =-\sum_{i}^{R}p_{i}\cdot \ln\left(p_{i}\right)$ where *R* represented the richness (total number of species identified in the sample) and *p_i_* represented the relative abundance of *i*^th^ species in the sample.

For multivariate analysis, data from all experiments (abundances of bacterial taxa, growth characteristics from calorimetry and metabolite productions/consumptions) was merged into a matrix table. Partial least squares discriminant analysis (PLS-DA) of the data was performed using web-based software MetaboAnalyst 3.0 (McGill University, Quebec, Canada) [28]. For in vitro and mice experiments results are expressed as mean ± SD. Comparisons were made using a Student’s *t*-test. Differences were considered as significant when *p* < 0.05 (*) or *p* < 0.01 (**) or *p* < 0.001 (***).

## 3. Results

### 3.1. ERK1/2 and mTOR Pathways Detection in Pancreatic Cancer Cells under ERS-Mimicking Culture Conditions

**Figure 1:**
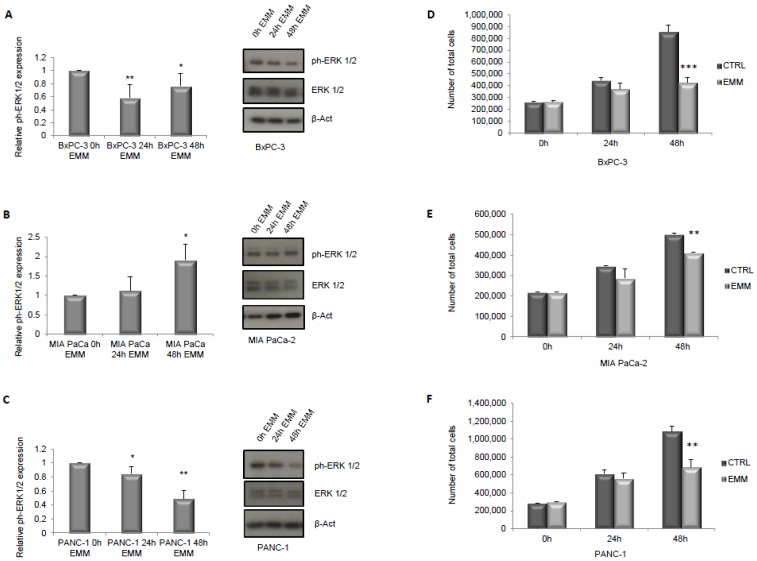
Immunoblot detection and quantification of relative phospho-ERK1/2 (extracellular signal-regulated kinase proteins) normalized to total ERK1/2 protein expression respectively in BxPC-3, MIA PaCa-2 and PANC-1 cells (**A**–**C**) treated with control medium (0 h EMM) or engineered resistant-starch (ERS)-mimicking medium (EMM) for 24 h (24 h EMM) or 48 h (48 h EMM). Cell count assay in BxPC-3, MIA PaCa-2 and PANC-1 cells grown in control medium (0 h EMM) or in ERS-mimicking medium for 24 h (24 h EMM) or 48 h (48 h EMM) (**D**–**F**). Results are expressed as means ± standard deviation (SD). Differences were considered as significant when *p* < 0.05 (*) or *p* < 0.01 (**) or *p* < 0.001 (***).

**Figure 2:**
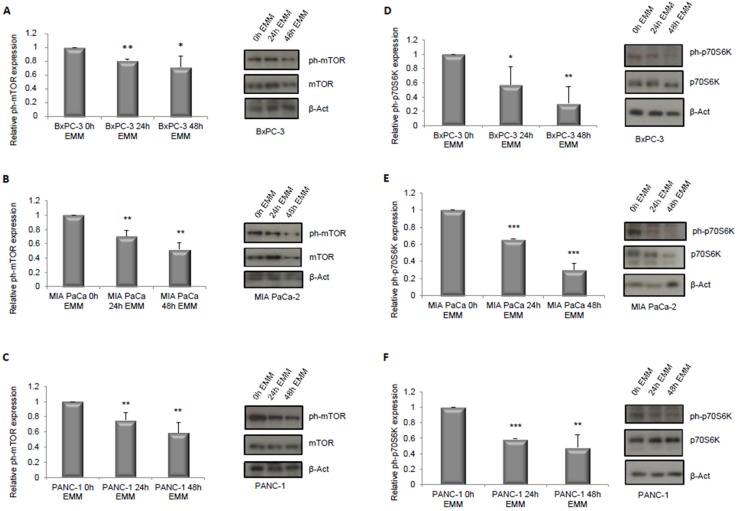
Immunoblot detection and quantification of relative phospho-mTOR (mammalian target of rapamycin) normalized to total mTOR protein expression respectively in BxPC-3, MIA PaCa-2 and PANC-1 cells (**A**–**C**) treated with control medium (0 h EMM) or ERS-mimicking medium for 24 h (24 h EMM) or 48 h (48 h EMM). Immunoblot detection and quantification of relative phospho-p70S6K normalized to total p70S6K protein expression respectively in BxPC-3, MIA PaCa-2 and PANC-1 cells (**D**–**F**) treated with control medium (0 h EMM) or ERS-mimicking medium for 24 h (24 h EMM) or 48 h (48 h EMM). Results are expressed as means ± SD. Differences were considered as significant when *p* < 0.05 (*) or *p* < 0.01 (**) or *p* < 0.001 (***).

As a first step, we hypothesized that the ERS-mimicking culture could be responsible for inhibiting the cells’ proliferation. To this aim, we assessed the activation of ERK1/2 and mTOR pathways, which are well known to be nutrient sensitive, to be involved in proliferation pathways [29,30,31], and specifically to be relevant for the growth of the cell lines used in our study [32,33,34,35]. As shown in Figure 1, culturing cells for both 24 h and 48 h in ERS-mimicking medium significantly inhibited ERK1/2 phosphorylation in Bx-PC3 and PANC-1 cell lines (A–C) while an increase in ERK1/2 phosphorylation level was observed in MIA PaCa-2 cells (B). The latter, however, showed a decreased level of ERK1/2 total form (B) by immunoblot analysis. Furthermore, all three cell lines showed a reduction in cell proliferation upon EMM treatment, as evaluated by the Muse Count & Viability assay, which reached statistical significance at 48 h (Figure 1D–F). Additionally, when the mTOR pathway was investigated, a reduced level of mTOR phosphorylation was observed in all three cell lines (Figure 2A–C) with a consequent reduction of its substrate p70S6K phosphorylation (Figure 2D–F), at both 24 and 48 h of EMM treatment.

### 3.2. Effect of ERS Diet on Pancreatic Cancer Xenograft Mice Tumor Growth

**Figure 3:**
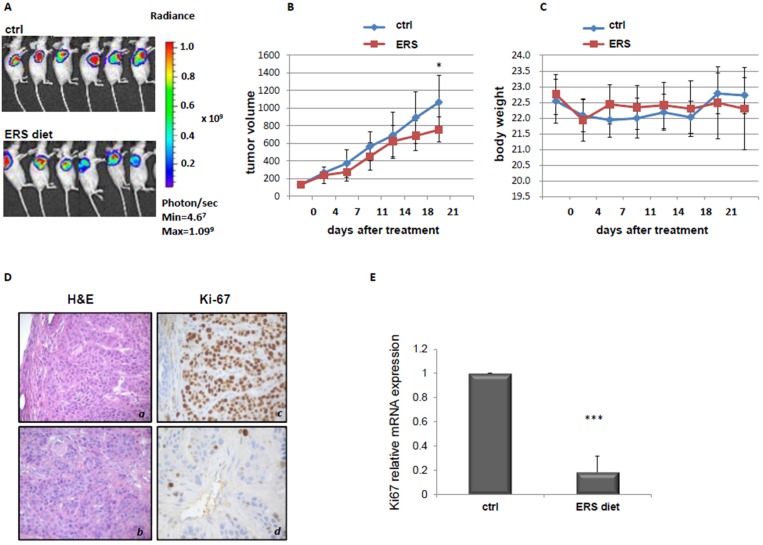
Effect of an ERS diet on PC tumor. BxPC-3-luc tumor-bearing nude mice were randomly assigned into two groups when tumor size reached an average volume of 100 mm^3^. Group 1 (standard diet), Group 2 (ERS diet). Bioluminescence signaling was measured as photons/sec (**A**). The tumor masses were harvested and tumor volume was evaluated (**B**). Body weight was also evaluated (**C**). H/E and Ki67 staining of PC biopsies of mice belonging to the two different groups (**D**). a, c—control diet, b, d—ERS diet. Ki67 mRNA expression in PC biopsies of mice fed with control (ctrl) or ERS diet, detected by quantitative real-time PCR (qRT-PCR) (**E**). *** *p* value < 0.001.

We then evaluated the effects of an ERS diet treatment in a xenograft pancreatic cancer mouse model. As shown in Figure 3A,B, mice subjected to the ERS diet displayed a slight but significant retarded progression of pancreatic cancer tumor (*p* = 0.04) as compared to control mice. No significant differences in total body weight were observed between the two mice groups (Figure 3C).

We then assessed the expression of proliferation and cell death/apoptosis markers in pancreatic cancer biopsies from mice. Immunohistochemistry revealed that Ki67 positivity was higher in mice fed with a control diet, with 60% of mice displaying the highest positive levels (Figure 3D panel *c*), while in the ERS diet group, 40% of mice were positive for Ki67 staining, while the remaining 60% of mice were mildly positive (panel *d*). Consistently, qRT-PCR showed a significant reduction in Ki67 mRNA expression in tumor biopsies of mice fed with an ERS diet, compared with mice fed with a control diet (Figure 3E). Additionally ERK1/2 and mTOR (with its direct substrates p70S6K) were determined by Western blot in a subset of mice treated with a control diet or an ERS diet. As shown in Supplementary Figure S1, both pathways tended to be down-regulated without reaching statistical significance.

### 3.3. Characterization of Microbiota and Metabolites of Fecal Samples

**Figure 4:**
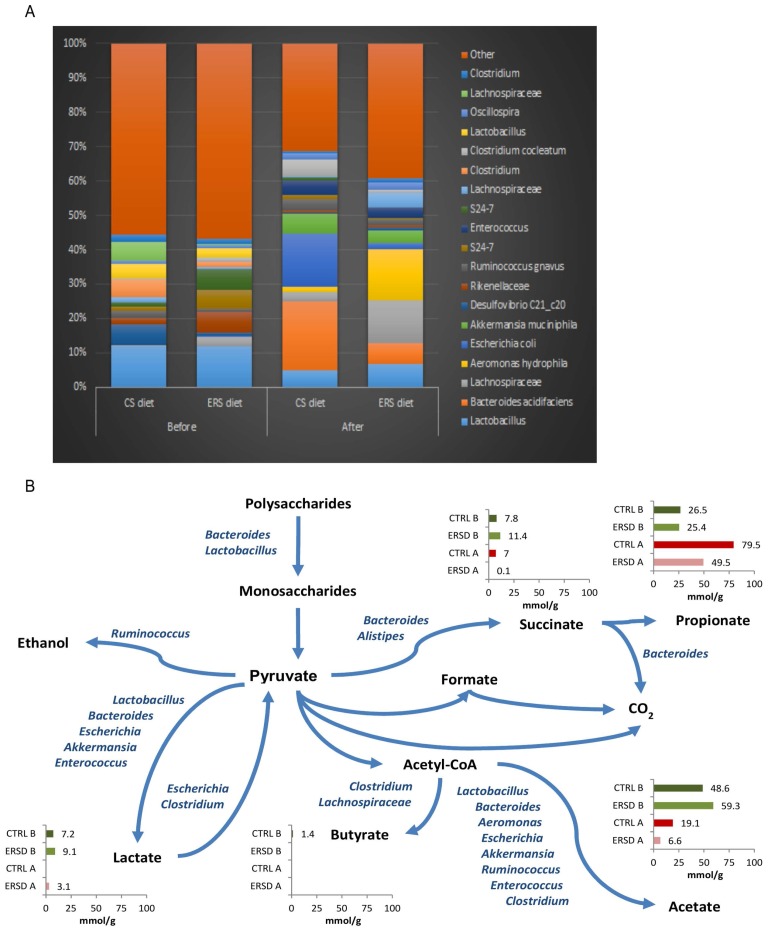
Composition of bacterial taxa in individual fecal samples. Panel (**A**) shows the most abundant 20 bacterial taxa of fecal samples, with an average abundance of at least 1% (average sum of reads in relative scale, %). Before and after indicate the time at which samples are taken in respect to cancer treatment; (**B**) Metabolic scheme and amount of organic acids in mouse fecal samples (mmol/g feces) before (**B**) and after (**A**) cancer induction. CTRL—control diet, ERS—resistant starch diet. Bacterial names on the pathway lines indicate the genera identified from the samples.

In total, 65 bacterial taxa that exceeded 0.5% relative abundance in feces of control and/or in ERS diet fed mice were found. The initial composition of fecal consortia was dominated by phylum *Firmicutes* (over 50%). The major taxa represented in the samples belonged to *Lactobacillus, Lachnospiraceae, Bacteroides, Blautia, Aeromonas and Escherichia* (Figure 4A). The majority of the detected bacteria were present in all fecal samples. However, diversity of microbiota was higher in the ERS diet fed mice than in the control group (Shannon indexes 3.56 ± 0.06 and 3.34 ± 0.2, respectively). After the cancer induction, diversity of microbiota decreased, especially between the control diet and the ERS diet (Shannon indexes 3.0 ± 0.01 and 3.05 ± 0.18, respectively). *Bacteroides acidifaciens* and *Esherichia* sp. were the dominant species (5%–20% and 6%–8%, respectively) in cancer-xenografted mice fed with the control diet, while species of *Blautia* and *Aeromonas* were dominant (over 15%) in cancer-xenografted mice fed with the ERS diet. *Bacteroides thetaiotaomicron* was found only in fecal samples of mice fed with an ERS diet and was present before, but not after, cancer induction (relative abundance of 1%). The latter was also detectable in minor amounts (0.1%), in xenografted mice fed with the control diet. Metabolite profiles differed between fecal samples depending on the nourishment type (control vs. ERS diet). The main fermentation product before cancer induction was acetate (53% and 56% from all acids produced on control or ERS diet, respectively), followed by propionate, succinate and lactate, while butyrate was detected only in negligible amounts (Figure 4B). After cancer induction, a significant reduction in acetate production was observed with both diets (2.5- and nine-fold on control and ERS diets, respectively), which was replaced by propionate production, especially in the control diet. It is remarkable that no lactate was produced under the control diet, while succinate production was negligible under the ERS diet. Total acid production before cancer induction was similar on both diets, however, after the induction it was almost two-fold reduced on the ERS diet from 105 to 59 mmol/g DW, but not on the control diet.

### 3.4. Growth Experiments with Fecal Microbiota

**Figure 5:**
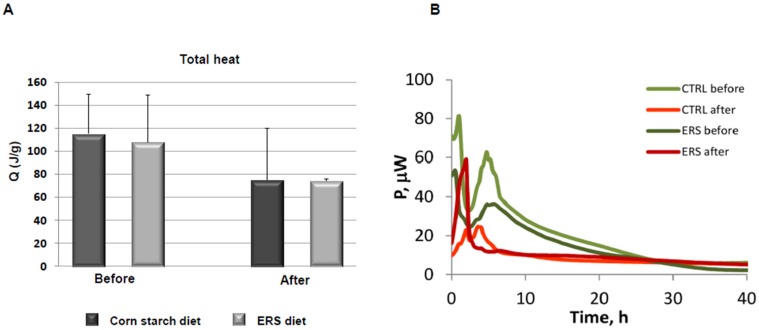
Total heat accumulated during the growth of fecal consortia in microcalorimetry experiments (**A**). Total emission of heat of mouse fecal sample was measured and normalized to sample weight. Before—before cancer treatment, After—after cancer treatment. Error bars represent SD; (**B**) Heat evolution patterns during the growth of fecal consortia in microcalorimeter without added substrate. “CTRL before” or “CTRL after” indicates the fecal sample of mice before or after cancer induction, respectively. ERS—resistant starch diet.

**Figure 6:**
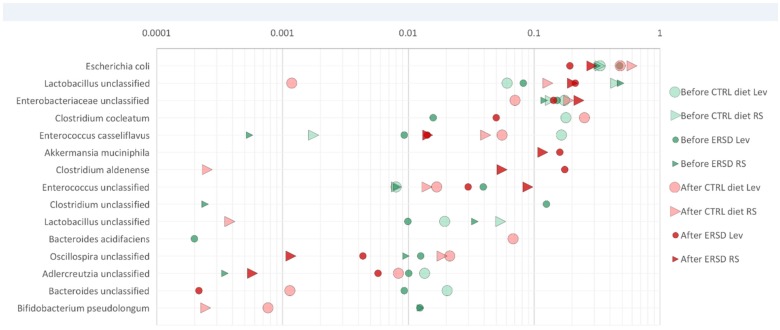
Enrichment of bacteria from mice fecal microbiota during the growth on RS and levan in microcalorimeter. The relative abundance of species or genera (family/order level if the genus not identified) assuming that 1 read = 1 cell at the end of growth in logarithmic scale. CTRL diet—control diet, ERS diet—resistant starch diet, “Before ERSD Lev” or “After ERSD Lev” indicates the fecal sample of mice before or after cancer induction, respectively, and growth of this consortia is monitored on levan in a microcalorimeter.

To elucidate the potential of the ERS diet and a polyfructan levan, for modulating the composition and fermentation pattern of the fecal microbiota, in vitro growth experiments were carried out. The growth experiments using fecal inocula from mice before and after cancer induction were performed in defined medium containing either levan, resistant starch, or no additional substrate (control). The heat generation (biomass growth) of the control cultures occurred in the residual substrates (complex carbohydrates and proteins) in the fecal material, accessible to microorganisms. Two phases could be discriminated on the growth curves from samples taken before the cancer induction (Figure 5B), but growth of bacteria from samples of cancer-induced mice fed with ERS medium was rapid within the single phase, indicating fast metabolic rates of bacteria in these samples. There was around a 30% difference in accumulated heat between samples collected before and after cancer induction (110–120 vs. 74–75 J/g, respectively, Figure 5A), independent of mice’ diets. The change of accumulated heat between samples collected before and after cancer induction, could indicate the modification of the diversity of microbial community (see above). Furthermore, important differences emerged between fecal microbiota growth from two diets on RS and levan in in vitro growth experiments. It was shown that both substrates (ERS and levan) supported the growth of *Escherichia, Lactobacillus, and Enterococcus* in fecal microbiota from mice fed with both diets (Figure 6). However, significantly higher relative amounts of *Lactobacillus* species were observed in pre-cancer fecal microbiota, especially from mice fed the ERS diet, grown on both levan as well as on RS (0.07 and 0.46, respectively). This was also in accordance with elevated lactate production compared to the growth on levan or control medium. It was also observed that the growth of *Escherichia* almost doubled in fecal samples from cancer-induced mice fed the control diet compared to the ERS diet. In contrast to resistant starch samples, levan-induced acetate production correlated with the increase of *Lactobacillus*, *Enterococcus* and *Escherichia* abundances in fecal microbiota samples, while *Clostridium cocleatum* became one of the dominant species in fecal cultures from mice fed with control diet. This species has been shown to increase resistance against colonization of potentially pathogenic *Peptoclostridium difficile* [36].

**Figure 7:**
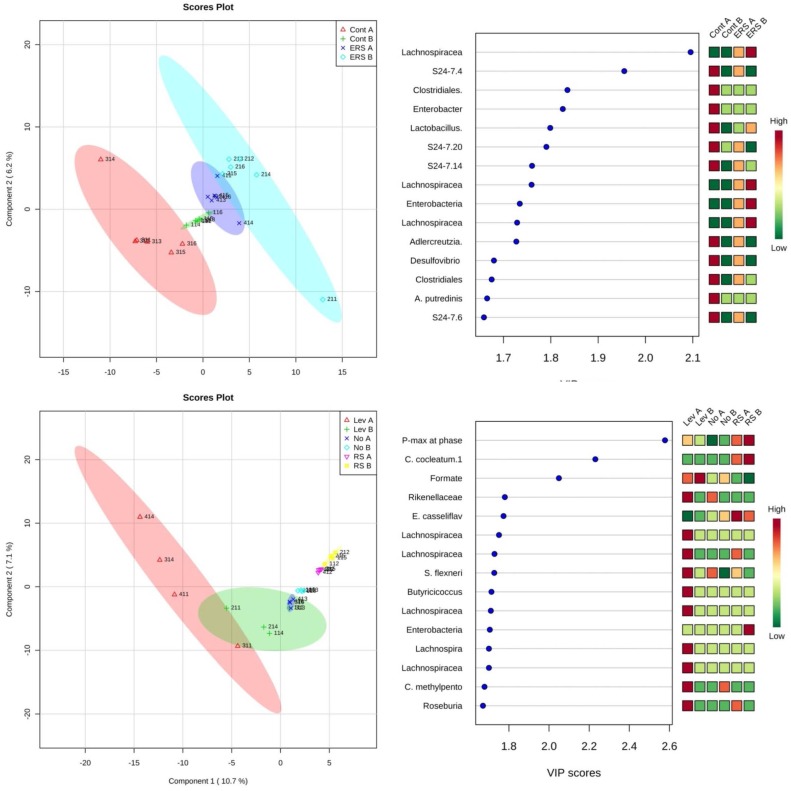
Score plots of Partial least squares discriminant analysis (PLS-DA) (**left**) and variable of importance (VIP) scores (**right**) grouped by substrates used in microcalorimetry. Plots derived from the integrated analysis of microcalorimetric data (total heat accumulated (Q), maximal heat evolution rate (Pmax), specific growth rate (μ), consumption of substrates (levan, amino acids) and formation of products (organic acids, gases and ethanol), and the bacterial genera grown in the microcalorimetry experiments. Analysis was done using the MetaboAnalyst 3.0 program [28]. Lev, NO and RS indicate levan, no added substrate, and resistant starch used in the microcalorimetry experiment, respectively. A and B in the names shows the samples taken before or after the induction of cancer, respectively.

By PLS-DA analysis, the combined data (metabolites, heat data and sequencing data) showed that maximal heat evolution rate, indicating rapid metabolic activity, was the most important parameter discriminating the growth of consortia in microcalorimetry experiments (Figure 7). Additionally two other important parameters having high variable of importance (VIP) scores were the relative abundance of *C. cocleatum*, which was present in high levels on RS, and formate production, which was highly produced on levan.

## 4. Discussion

It is now accepted that dietary restriction has beneficial health effects, including increased lifespan and cancer prevention [37]. Recent studies by our group and others revealed an association between caloric restriction achieved with fasting, and better response to chemotherapy in certain kinds of cancer [5,22,38], including pancreatic cancer [5], as demonstrated both in vitro and in animal models. This dietary intervention may provide beneficial effects for human cancers too, but the difficulty for the patient to in accepting not to eat, and the potential worsening of the cancer-related weight loss due to fasting make the adoption of new approaches necessary.

In the current study we assessed whether an engineered diet replacing corn starch with resistant starch could be a valid alternative to fasting, in counteracting pancreatic cancer. First of all, we assessed the in vitro effects of an ERS-mimicking medium, in which reduction of glucose content from 2 to 0.5 g/L mimicked the decreased intestinal release of glucose due to indigestible resistant starch. All three pancreatic cancer cell lines used, BxPC-3, MIA PaCa-2 and PANC-1 showed a significant decrease in proliferation rate upon EMM, as measured by Muse Count & Viability assay. Consistent results were obtained in vivo, where xenografted mice fed an ERS diet showed significant retardation in tumor growth as compared to mice fed a control diet. These results are supported by the existing literature concerning the protective role of resistant starch consumption toward colorectal [39,40,41] and breast cancer [42,43].

Since it is known that intestinal microbiota can be easily manipulated by a diet that selectively enriches specific microbial groups [12,13,14], and that changes in gut microflora can affect the development of several diseases [15,16,17,18] including pancreatic cancer [21], we then investigated the effects of an ERS diet on the composition and metabolism of mouse fecal microbiota.

In cancer-induced mice, the control diet stimulated the growth of *Bacteroides acidifaciens, Akkermansia muciniphila, Ruminococcus gnavus, Clostridium cocleatum* and *Escherichia*, which might be the cause of inflammation, as it has been shown that *Bacteroides acidifaciens* is associated with gut inflammation-colitis in murine gut [44]. Similarly Png et al. [45] showed that the amount of *R. gnavus* increased 4-fold in case of inflammatory bowel disease. The proteobacteria *Escherichia coli* and *Aeromonas* have been linked to lipopolysaccharide- (LPS) driven inflammatory interleukin (IL) activation [46,47]. In the current study, after cancer induction, the abundance of proteobacteria increased in both cases (from 6% to 17% on the control diet, and from 3% to 17% on the RS diet); however, *Escherichia* was the dominant genus in the control diet, while *Aeromonas* was dominant on the RS diet. Inflammation by *Aeromonas hydrophila* has been described in mice [47,48]. Ko et al. [47] observed that the *Aeromonas* strain caused significantly higher serum levels of IL-1beta and IL-6. However, with the ERS diet, around half the inflammation-associated bacteria were detected as compared to control diet. Pro-inflammatory microorganisms such us *B. acidifaciens*, *E. coli*, *R. gnavus* and *Clostridium cocleatum* were significantly reduced with the ERS diet (relative abundance of the sum of these bacteria were 0.18 vs. 0.4, Figure 4A). Moreover, in our study, overgrowth of *Escherichia* (on the control diet) was accompanied by mucin degrading bacteria. Among active mucin degraders *Akkermansia muciniphila, Clostridium cocleatum* and *Bacteroides acidifaciens* were observed*.* This indicates that significant amount of *B. acidifaciens* in the feces of mice (17% in our study) might be related to the inflammatory response of pancreatic cancer. Overall, the ERS diet modulated gut microbiota composition, especially affecting bacterial populations involved in inflammation. Since pancreatic cancer is a kind of tumor whose development is strongly driven by inflammation [21,49], one might speculate that ERS diet could have influenced pancreatic tumor growth by perturbing microbial communities sustaining inflammation.

The normal healthy murine microbiota consists of 1:1 to 1:2 *Bacteroidetes* to *Firmicutes*, of which more than 10% are lactobacilli. As shown in this study, the abundance of lactobacilli decreased from 17% to 5%–7% after cancer induction, indicating inflammation driven changes. These changes led to modifications in metabolite profiles. For example, the ratio of acetate to lactate fell from 6.5–7 before to about 2 after cancer induction, whilst no lactate was detected on the control diet after cancer induction. At the same time, the ratio of acetate to propionate changed from 1.8–2.3 to 0.13–0.24, which could be explained by an overgrowth of propionic acid producers *B. acidifaciens* and *A. muciniphila*. Decreased acetate to propionate ratio (1.1) has been observed also in an experiment where rats were fed an inulin-enriched diet, while on ordinary starch diet, the ratio was 2.8 [50].

Of note, even though butyrate was below the level of detection, a remarkable increase (17% vs. 3%) in *Lachnospiraceae*, potentially harboring butyrate producers, was observed in mice fed the ERS diet compared to mice fed the control diet after cancer induction. Butyrate has been proven to inhibit proliferation, and to promote differentiation and apoptosis in different cancer cell lines [51,52,53,54,55,56], including pancreatic lines [57,58]. Furthermore, butyrate inhibits pancreatic cancer invasion [59].

In microcalorimetry experiments, the organic acids (lactic and acetic acids) and amount of lactobacilli were used as discriminating parameters between substrates. During the growth of fecal microbiota on levan-containing medium, lactic and acetic acids were produced in equimolar amounts, while on RS mostly lactic acid was produced. Proportions of *Lactobacilli* and *Escherichia* in fecal microbiota were 0.45 and 0.33 when grown on RS, and 0.07 and 0.4 in the case of growth on levan. Our experiments showed that colon microbiota can be specifically modulated by different substrates such as RS and levan, and hence these should have an effect also on tumor growth in vivo; this should be analyzed in further experiments.

The beneficial effects of the ERS diet observed in our study are encouraging, and raise the question of whether similar results would be obtained in translating this food regimen to humans. To date, resistant starches as food ingredients for humans are considered generally safe and potentially beneficial [60]. The main health benefits associated to RS intake are: (a) the lowering of glycaemia with consequent amelioration of diabetes and insulin resistance, (b) lower caloric intake, which would reduce body weight and obesity, and (c) a protective role against intestinal inflammation and cancer, which is mainly attributable to modification in microbiota and butyrate production [60,61]. No harmful effects of RS have been recognized thus far, except for some gastrointestinal symptoms due to excessive intake [60]. When translating this approach from animal to human populations, however, some limitations should be taken into account. While some kinds of resistant starch are naturally present in many foods, others have to be artificially added, which has been already successfully achieved in a variety of food products [60]. Nevertheless, adding resistant starch may alter the rheological and organoleptic properties of the food [11,60], although the food should maintain acceptable sensory characteristics. Finally, it must be considered that supportive treatment with a resistant starch diet in human cancers may require periods longer than those used in our animal study, so that the consequences of long-term consumption of such a diet should be carefully evaluated. To date, however, a positive feedback has been obtained from a study assessing the effects of a long-term intake of resistant starch in pigs: better mucosa integrity, decreased colonic cell apoptosis, and reduced colonic and systemic immune reactivity have been observed [61].

## 5. Conclusions

In the current study, ERS-mimicking culture conditions have shown to impair the proliferation of three PC cell lines, through the inactivation of two signaling pathways known to be nutrient-sensitive. Moreover, the ERS diet was found to influence the composition and metabolism of the gut microbiota, and this was paralleled by the retardation of tumor growth in the PC xenograft mouse model. Although further experiments are needed to elucidate the mechanisms underlying this phenomenon, an interesting reduction in pro-inflammatory bacterial populations suggests that a further in vivo effect of ERS diet may be the reduction of inflammation. Overall, our results suggest that dietary interventions replacing corn starch with resistant starch could be adopted in support of conventional therapies, in the clinical management of pancreatic cancer.

## Supplementary Materials

Supplementary materials can be found at http://www.mdpi.com/2072-6643/9/4/331/s1. Figure S1: Immunoblot detecion of relative phospho-ERK1/2 normalized to total ERK1/2 protein expression (*p* = 0.098) (A) in control xenograft PC mice’ biospies (ctrl) and mice fed with ERS diet. Phosphorylation levels of mTOR (*p* = 0.688) and its substrate p70S6K (*p* = 0.359) (B) detected by immunoblot in control xenograft PC mice’ biospies (ctrl) and mice fed with ERS diet (ERSD).
