# Supplementary material for: Engineered Resistant-Starch (ERS) Diet Shapes Colon Microbiota Profile in Parallel with the Retardation of Tumor Growth in In Vitro and In Vivo Pancreatic Cancer Models
Source: Nutrients. 2017 Mar 27;9(4):331. doi: 10.3390/nu9040331 (PMC5409670; doi:10.3390/nu9040331)
Supplement: Supplementary file 1 [file nutrients-09-00331-s001.docx]

Supplementary Materials: Engineered Resistant-Starch (ERS) Diet Shapes Colon Microbiota Profile in Parallel with the Retard of Tumor Growth in In Vitro and In Vivo Pancreatic Cancer Models

Concetta Panebianco, Kaarel Adamberg, Signe Adamberg, Chiara Saracino, Madis Jaagura, Kaia Kolk, Anna Grazia Di Chio, Paolo Graziano, Raivo Vilu and Valerio Pazienza

**Table S1.** Production of organic acids during the growth of fecal microbiota in microcalorimeter without added substrate (mmol/gDW).

| **Sample** | **Acetate** | **Butyrate** | **Formate** | **Lactate** | **Propionate** | **Succinate** |
| --- | --- | --- | --- | --- | --- | --- |
| Control diet before | 906 ± 160 | 100 ± 23 | 335 ± 248 | 199 ± 179 | 185 ± 50 | 143 ± 52 |
| Control diet after | 559 ± 3 |  | 260 ± 39 | 51 ± 30 | 133 ± 27 | 74 ± 3 |
| ERS diet before | 922 ± 110 | 33 ± 46 | 353 ± 246 | 160 ± 26 | 88 ± 124 | 131 ± 22 |
| ERS diet after | 533 ± 211 |  | 338 ± 27 | 220 ± 56 | 73 ± 5 | 65 ± 58 |


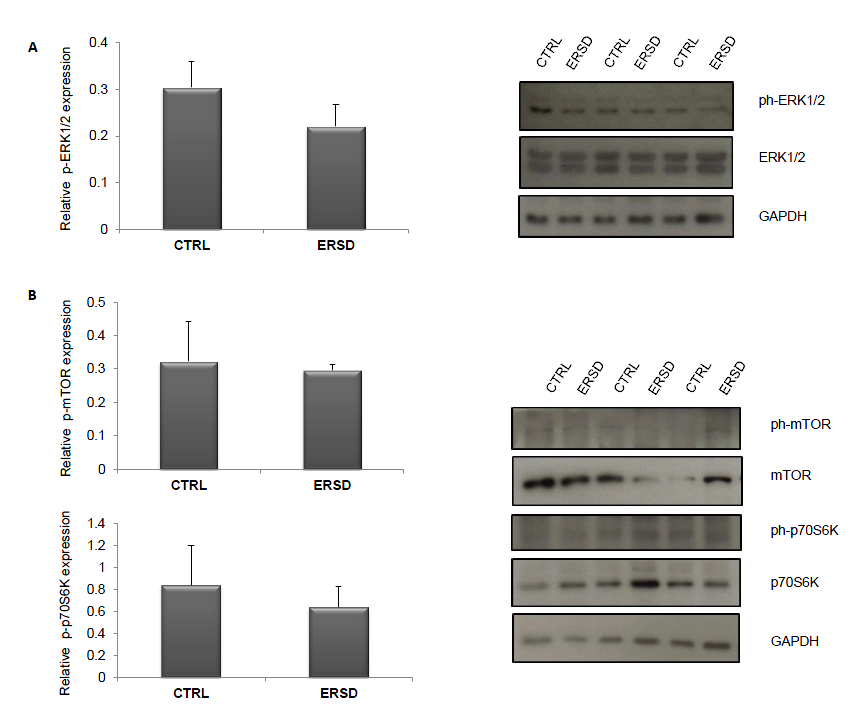


**Figure S1.** Immunoblot detecion of relative phospho-ERK1/2 normalized to total ERK1/2 protein expression (*p* = 0.098) (**A**) in control xenograft PC mice’ biospies (ctrl) and mice fed with ERS diet. Phosphorylation levels of mTOR (*p* = 0.688) and its substrate p70S6K (*p* = 0.359) (**B**) detected by immunoblot in control xenograft PC mice’ biospies (ctrl) and mice fed with ERS diet (ERSD).
